# Supplementary material for: Saccharomyces boulardii (CNCM I-745) improves intestinal damage in sepsis by remodeling intestinal flora structure
Source: Front Cell Infect Microbiol. 2025 Jul 31;15:1602792. doi: 10.3389/fcimb.2025.1602792 (PMC12350329; doi:10.3389/fcimb.2025.1602792)
Supplement: Supplementary file 1 [file DataSheet1.zip › summary/summary/6_Taxonomy_community/3_Circos_plot/6_Genus/Genus_Circos_top5_Group.pdf]

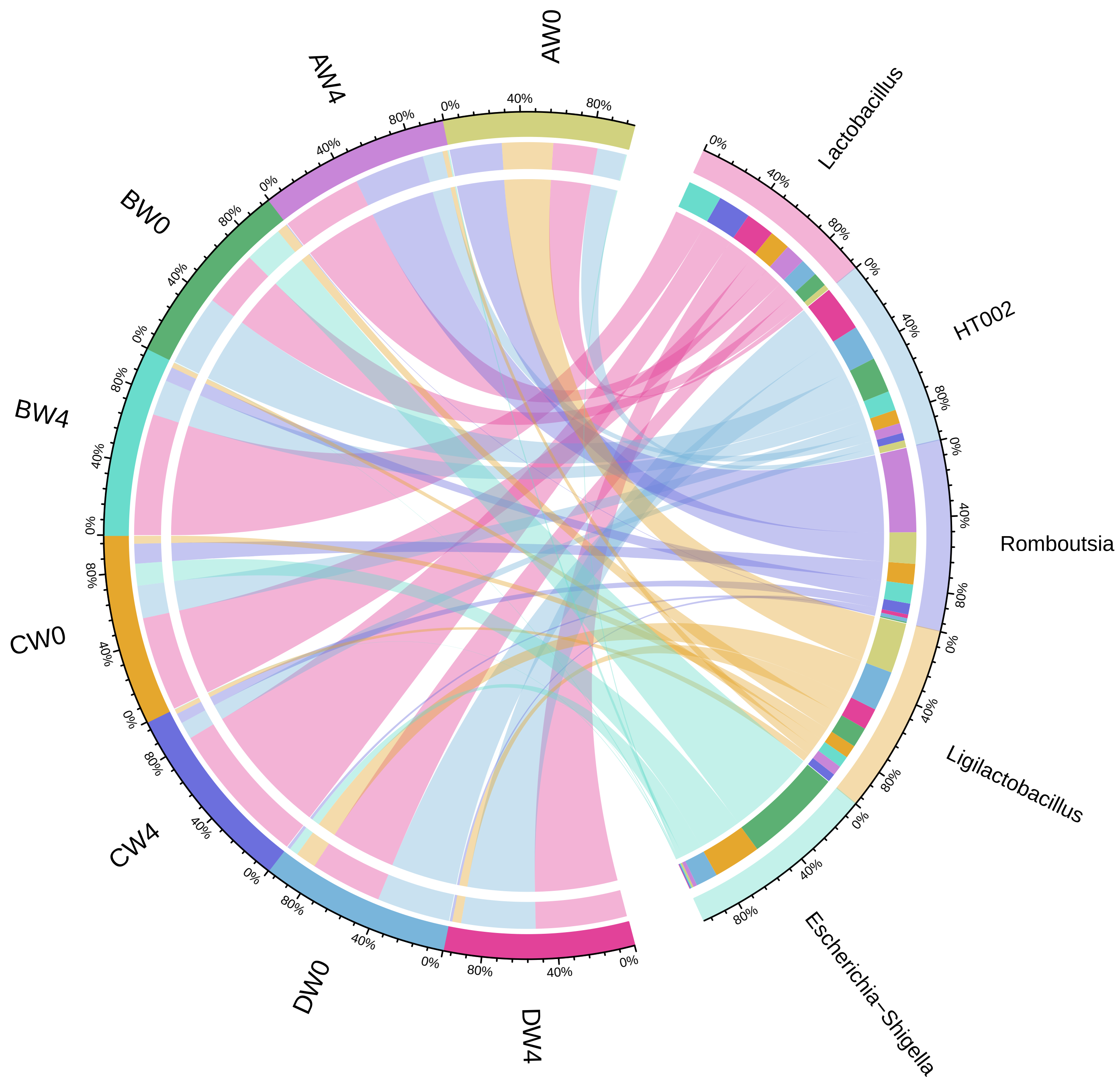

AW0
AW4
BW0
BW4
CW0
CW4
DW0
DW4
Lactobacillus
HT002
Romboutsia
Ligilactobacillus
Escherichia–Shigella
0%
40%
80%
